# 92438 Symptom Dynamics and Biomarkers of Disease Progression in Older Adult Patient-Caregiver Dyads During Care Transitions after Heart Failure Hospitalization: Study Design and Anticipated Results

**DOI:** 10.1017/cts.2021.732

**Published:** 2021-03-30

**Authors:** Julie T. Bidwell, Emilio Ferrer, Christopher S. Lee, Martin Cadeiras, Karen S. Lyons, Heather M. Young, Ladson Hinton

**Affiliations:** 1 Betty Irene Moore School of Nursing; 2 University of California Davis; 3 Connell School of Nursing, Boston College; 4 UC Davis Health

## Abstract

ABSTRACT IMPACT: This study is designed to address a critical gap in our understanding of how aging patients and caregivers recognize and respond to clinically important changes in heart failure symptoms during vulnerable transitions. OBJECTIVES/GOALS: Research on family involvement in heart failure (HF) symptom response is limited. Our objective is to examine HF symptom monitoring processes in couples after HF hospitalization, and quantify how coupled symptom assessments predict symptom response, patient clinical events, care strain, and dyad health during the high-risk post-discharge period. METHODS/STUDY POPULATION: This is an ongoing T2 translational study that employs an intensive longitudinal design. Adults aged ≥65 years hospitalized for HF and their caregiving spouse/partner are enrolled. The target n is 48 dyads. Over 5 weeks of follow-up, dyads complete daily diaries assessing patient HF symptoms. Clinical biomarkers of HF severity (NTproBNP, ST2) are also collected. Primary study endpoints are dyads’ HF symptom response behaviors and caregiver strain; secondary endpoints are dyads’ health status and patient clinical events. Dyadic dynamics of symptom assessment will first be characterized using dyadic autoregressive time series models. Subsequently, we will extract cross-partner effect parameters from the time series models and test whether dyadic effects predict the trajectories of each of our endpoints. RESULTS/ANTICIPATED RESULTS: This study is currently underway. In line with our study hypotheses, we anticipate that couples who assess patient symptoms similarly (dyadic agreement), and whose symptom assessments accurately reflect clinical severity, will be more likely to respond to symptoms appropriately with lower stress to the caregiving partner, and have better trajectories of health (self-reported and clinical). Characterizing dyadic symptom dynamics will provide important insight into the day-to-day process of symptom recognition in couples. Further, quantifying dyadic symptom dynamics in relation to our endpoints will provide information on the clinical value of dyadic symptom agreement, and whether it might be a target for future interventions to support better symptom response and health outcomes for both dyad members. DISCUSSION/SIGNIFICANCE OF FINDINGS: This project innovates on existing paradigms by applying family-level theory and techniques to better understand and support interventions for couples during post-discharge HF transitions - a vulnerable period for older adults that has traditionally been studied almost exclusively at the patient-level, with marginal success.

